# The Potential of *Streptomyces* as Biocontrol Agents against the Rice Blast Fungus, *Magnaporthe oryzae* (*Pyricularia oryzae*)

**DOI:** 10.3389/fmicb.2017.00003

**Published:** 2017-01-17

**Authors:** Jodi Woan-Fei Law, Hooi-Leng Ser, Tahir M. Khan, Lay-Hong Chuah, Priyia Pusparajah, Kok-Gan Chan, Bey-Hing Goh, Learn-Han Lee

**Affiliations:** ^1^Novel Bacteria and Drug Discovery Research Group, School of Pharmacy, Monash University MalaysiaBandar Sunway, Malaysia; ^2^Department of Pharmacy, Absyn University PeshawarPeshawar, Pakistan; ^3^Biomedical Research Laboratory, Jeffrey Cheah School of Medicine and Health Sciences, Monash University MalaysiaBandar Sunway, Malaysia; ^4^Division of Genetics and Molecular Biology, Faculty of Science, Institute of Biological Sciences, University of MalayaKuala Lumpur, Malaysia; ^5^Center of Health Outcomes Research and Therapeutic Safety, School of Pharmaceutical Sciences, University of PhayaoPhayao, Thailand

**Keywords:** *Streptomyces*, biocontrol, antifungal, rice, disease

## Abstract

Rice is a staple food source for more than three billion people worldwide. However, rice is vulnerable to diseases, the most destructive among them being rice blast, which is caused by the fungus *Magnaporthe oryzae* (anamorph *Pyricularia oryzae*). This fungus attacks rice plants at all stages of development, causing annual losses of approximately 10–30% in various rice producing regions. Synthetic fungicides are often able to effectively control plant diseases, but some fungicides result in serious environmental and health problems. Therefore, there is growing interest in discovering and developing new, improved fungicides based on natural products as well as introducing alternative measures such as biocontrol agents to manage plant diseases. *Streptomyce*s bacteria appear to be promising biocontrol agents against a wide range of phytopathogenic fungi, which is not surprising given their ability to produce various bioactive compounds. This review provides insight into the biocontrol potential of *Streptomyces* against the rice blast fungus, *M. oryzae*. The ability of various S*treptomyces* spp. to act as biocontrol agents of rice blast disease has been studied by researchers under both laboratory and greenhouse/growth chamber conditions. Laboratory studies have shown that *Streptomyces* exhibit inhibitory activity against *M. oryzae*. In greenhouse studies, infected rice seedlings treated with *Streptomyces* resulted in up to 88.3% disease reduction of rice blast. Studies clearly show that *Streptomyces* spp. have the potential to be used as highly effective biocontrol agents against rice blast disease; however, the efficacy of any biocontrol agent may be affected by several factors including environmental conditions and methods of application. In order to fully exploit their potential, further studies on the isolation, formulation and application methods of *Streptomyces* along with field experiments are required to establish them as effective biocontrol agents.

## Introduction

Rice (*Oryza sativa*) is an important food crop and it is the staple diet of over three billion people around the world, particularly in Asia^1^ (Abdullah et al., 2006; Skamnioti and Gurr, 2009; Hosseyni-Moghaddam and Soltani, 2013). Nearly half of Asia’s population depends on rice as their main food source, making Asia the region with the highest rice consumption—more than 110 kg per capita annually (Hosseyni-Moghaddam and Soltani, 2013; Muthayya et al., 2014). Rice is grown in more than a 100 countries across a wide range of climatic conditions, ranging from rivers deltas to mountainous regions (Kyndt et al., 2014). Asian countries including China, India, Indonesia, Thailand, Philippines, Vietnam, Bangladesh, and Myanmar account for approximately 90% of the world’s total rice production (Khush, 2005; Abdullah et al., 2006; Muthayya et al., 2014). The other rice-producing countries include Egypt, Brazil, sub-Saharan countries, and the USA (Kyndt et al., 2014). Wherever it is grown, however, rice is susceptible to diseases with far reaching economic implications. Infection with phytopathogenic fungi are among the most worrying of these diseases as it may result in significant crop yield losses, and additionally, some of the fungi produce compounds which are potentially toxic upon consumption (Chaiharn et al., 2009; Suprapta, 2012). For instance, several *Fusarium, Aspergillus*, and *Penicillium* species are capable of producing mycotoxins (e.g., aflatoxins, citrinin, fumonisins, ochratoxin A, and zearalenone) which can be harmful to human beings if they are ingested via consumption of contaminated rice (Almaguer et al., 2012; Ferre, 2016). Given that the present methods of preventing rice diseases are not entirely satisfactory from several angles, it is imperative to seek new and effective methods of prevention in order to produce rice that is safe for consumption as well as to reduce crop yield losses.

Currently, *Magnaporthe oryzae* (anamorph *Pyricularia oryzae*) is regarded as one of the most important phytopathogenic fungi as it is the causal agent of rice blast— the most destructive disease of rice (Dean et al., 2012). *M. oryzae* B. Couch (anamorph *Pyricularia oryzae* Cav.), also known as *Magnaporthe grisea* (Hebert) Barr (anamorph *Pyricularia grisea* Sacc.), is a haploid filamentous ascomycete fungus (Bussaban et al., 2005). *M. oryzae* is defined as a new species distinct from *M. grisea* based on multilocus gene genealogy and laboratory mating experiments by Couch and Kohn (2002). On the basis of phylogenetic analysis, *Magnaporthe* is now separated into two distinct clades—one clade associated with *Digitaria* (crabgrass) maintains the name *M. grisea*, while the other clade associated with rice and other cultivated grasses was characterized as a novel species and given the name *M. oryzae*. Given the phylogenetic differences, however, there are no morphological differences between the isolates from these two clades. As a result, the names *M. oryzae* and *M. grisea* are still used interchangeably by scientists for the fungal isolates that infect rice (Besi et al., 2009; Wilson and Talbot, 2009).

*Magnaporthe oryzae* infects the aerial parts of the rice plant — including leaves, nodes, stems, and panicles— at all stages of development (Wilson and Talbot, 2009). Infection results in rice blast symptoms such as leaf blast, node blast, collar rot, neck rot, and panicle blast; this generally manifests as purplish/grayish/brownish/whitish spots or lesions as well as withering of leaves (Kato, 2001). This fungus was later discovered to also have the ability to infect the roots of the rice plant; and infection of the root may eventually spread to the aerial tissues, causing rice blast disease (Dufresne and Osbourn, 2001; Sesma and Osbourn, 2004). However, the exact nature and sequence of the process by which rice blast infects the roots of the rice plant has yet to be fully eludicated (Sesma and Osbourn, 2004). Rice blast disease has been reported in approximately 85 countries, mainly in Asia, Africa, and Latin America (Kato, 2001; Besi et al., 2009). Yield loss due to rice blast ranges from approximately 10–30% annually in the various rice producing countries and can reach up to 50% during disease epidemics (Skamnioti and Gurr, 2009; Ashkani et al., 2015).

Efforts have been made by researchers to identify and analyze the avirulence (*AVR*) genes of *M. oryzae.* This would serve as the basis of understanding fungal mechanisms of pathogenesis and clarifying the mechanisms responsible for the coevolution of fungal effectors and their host targets (Yoshida et al., 2009). Besides this, development of cultivar-specific resistance involving gene-for-gene system can be achieved by conditioning resistance through high-yielding rice cultivars carrying single dominant disease resistance (*R*) genes to a single corresponding dominant *AVR* gene in a particular pathogen strain (Skamnioti and Gurr, 2009). To date, four *AVR* genes have been isolated from *M. oryzae*: *PWL1* and *PWL2* genes which encode Gly-rich hydrophobic proteins with secretion signal sequences, *AVR-Pita* gene which encodes a putative secreted protein with similarity to metalloproteases, and *ACE1* gene which encodes a putative hybrid protein of a polyketide synthase and a peptide synthase (Skamnioti and Gurr, 2009; Yoshida et al., 2009). In contrast, more than 25 resistance (*R*) genes encoding proteins that recognize *M. oryzae* AVRs have been mapped on the rice genome (Yoshida et al., 2009).

Besides breeding of cultivar-specific resistance, several control methods have been attempted to manage plant diseases. Among these, chemical control is the most commonly used method yielding effective management of plant diseases (Hirooka and Ishii, 2013). With reference to control of rice blast, chemical control involves the use of pesticides, specifically fungicides (Skamnioti and Gurr, 2009). A number of fungicides have been used against this disease, for instance, azoxystrobin, benomyl, carbendazim, carpropamid, dithiocarbamate, edifenphose, fenoxanil, tiadinil, tricyclazole, pyroquilon, probenazole, iprobenfos, isoprothiolane, metominostrobin, and propiconazole (Kato, 2001; Skamnioti and Gurr, 2009; Pooja and Katoch, 2014). Generally, the effectiveness of fungicides depends on several factors: the compound itself, the timing and method of application, the level of disease present, the efficiency of disease forecasting systems, and the rate of emergence of fungicide resistant strains (Kato, 2001; Skamnioti and Gurr, 2009). Although they are effective at controlling the fungal infections in rice, there are growing public concerns over the use of synthetic fungicides. Misuse and excessive use of synthetic pesticides (e.g., fungicides, insecticides, and herbicides) might cause environmental pollution, residual toxicity, development of pesticide resistance, reduce soil quality, and damage to natural ecosystems (Pimentel et al., 1991; Komárek et al., 2010; Suprapta, 2012; Yoon et al., 2013). Furthermore, human exposure to pesticides may cause poisoning and harmful side-effects to organs and/or biological processes (Fattahi et al., 2015). Pesticide poisoning is a significant occupational health issue in developing countries, likely due to insufficient or poor occupational safety practices. Appropriate work practices are essential to ensure the safety of workers, particularly in the case of agricultural workers who are often exposed to pesticides during application and handling operations such as mixing, cleaning, loading spray equipment, and disposing of empty containers (Kesavachandran et al., 2009). Side effects and symptoms caused by exposure to pesticides have been reported in several developing countries. For instance, farmers in Vietnam reported symptoms such as skin irritation, headache, dizziness, eye irritation, shortness of breath, and acetyl cholinesterase inhibition due to the exposure to various pesticides during mixing and spraying (Dasgupta et al., 2007).

New and improved fungicides with minimal side effects are required in order to prevent these concerns. Nowadays, natural products which are safe for the environment and have low toxicity to living organisms are gaining interest as important sources for the development of fungicides, and these may serve as effective substitutes for synthetic fungicides (Martínez, 2012; Yoon et al., 2013). Moreover, another alternative approach to the use of fungicides is the use of microbial antagonists as biocontrol agents (Suprapta, 2012). Biocontrol agents are microorganisms that suppress plant pathogens (Pal and Gardener, 2006); they can achieve biological control through competition, antibiosis, and hyperparasitism (Montesinos, 2003). Biological control of plant diseases is known to be more cost effective, safe and environmentally friendly as compared to the use of fungicides. *Streptomyces* bacteria are among the microbial antagonists that have been exploited for the biological control of plant diseases. This review aims to encapsulate the current body of knowledge of the biocontrol potential of *Streptomyces* against the rice blast fungus, *M. oryzae*.

## *Streptomyces* spp. As Biocontrol Agents Against *M. oryzae*

*Streptomyces* is the largest genus of the phylum *Actinobacteria* and was first proposed by Waksman and Henrici (1943). The genus *Streptomyces* consists of a group of Gram-positive, aerobic, non-motile, catalase positive, and non-acid-fast bacteria with a filamentous form that resembles fungi (Flärdh and Buttner, 2009; Hasani et al., 2014). Currently, over 700 species of *Streptomyces* have been identified^2^ and these bacteria have relatively large genomes of approximately 8–9 Mbp in size with a high GC content of more than 70% (Wu et al., 2005; Hasani et al., 2014; Ser et al., 2015c).

The members of *Streptomyces* are well-known for their ability to produce a variety of bioactive compounds with different bioactivities such as antibacterial (Schumacher et al., 2003; Ramesh and Mathivanan, 2009; de Lima Procópio et al., 2012; Kumar et al., 2014; Lee et al., 2014a,b; Ser et al., 2016a), antifungal (Lam, 2006), antiviral (Ara et al., 2014), immunosuppressive (Kino et al., 1987), anticancer, and antioxidant properties (Ser et al., 2015a; Tan et al., 2015, 2016). These bioactive compounds have important applications in various fields. For example, approximately 75% of commercially useful antibiotics were derived from the genus *Streptomyces* and they are thus the primary antibiotic-producing organisms exploited by the pharmaceutical industry (Pelaez, 2006; Lee et al., 2014b; Kashif et al., 2016). *Streptomyces* strains also have important applications in the agricultural field through their biological control potential against phytopathogens, particularly phytopathogenic fungi (Gonzalez-Franco and Robles-Hernandez, 2009). In line with this, research on the biological control of rice blast disease using different *Streptomyces* species has been conducted under laboratory conditions, as well as in greenhouse or growth chamber conditions.

### *In vitro* Experiments

Studies have reported *in vitro* antagonism of different *Streptomyces* spp. against *M. oryzae* which were tested using the dual culture method. Dual culture method has been widely used for preliminary screening of biocontrol agents including fungi and bacteria. This method allows the biocontrol agent and pathogen to interact on a solid medium in a Petri dish, under optimal conditions for both organisms. The degree of inhibition is recorded by observing the inhibition zone produced or the overgrowth of the pathogen by the biocontrol agent (Desai et al., 2002).

In a study conducted by Ningthoujam et al. (2009), *S*. *vinaceusdrappus* isolated from Loktak lake sediment was first reported to exhibit antagonistic activity against *M. oryzae* (*P. oryzae* MTCC 1477). *S. vinaceusdrappus* also showed maximal antagonistic activity against most of the rice fungal pathogens tested, which include *Curvularia oryzae, Bipolaris oryzae*, and *Fusarium oxysporum*. *S. vinaceusdrappus* inhibited the mycelial growth of *P. oryzae* by 53.5%, which was relatively good since more than 50% of the mycelial growth was inhibited. Boukaew and Prasertsan (2014) reported that *Streptomyces philanthi* RM-1-138 isolated from rhizosphere soil of chili pepper in Southern Thailand exhibited significant antifungal activity against *M. oryzae* (*P. oryzae* PTRRC-18), with 88.73% inhibition of mycelial growth of the rice blast fungus. This suggests that *S. philanthi* RM-1-138 has greater inhibition against the rice blast fungus as compared to *S. vinaceusdrappus*.

The *in vitro* assay conducted by Li et al. (2011) showed that *Streptomyces globisporus* JK-1 demonstrated the most pronounced inhibitory effects against *M. oryzae* as compared to other phytopathogenic fungi tested in the study. *S. globisporus* JK-1 inhibited mycelial growth of *M. oryzae* with an inhibition zone of 15 mm out of 35 mm. A study also showed that endophytic *Streptomyces* from rice cultivars in China demonstrate antagonism against rice fungal pathogens, particularly 54.5% of *Streptomyces griseofuscus* and 21.8% of *Streptomyces hygroscopicus* were the most active among the studied population of antagonistic endophytic *Streptomyces* which exhibited strong antagonism against *M. oryzae* (Tian et al., 2004). *Streptomyces sindeneusis* isolate 263 and *Streptomyces* isolate 339 obtained from agricultural soils of Kerman in Iran were also found to inhibit *M. oryzae* (Zarandi et al., 2009, 2013). In addition, Khalil et al. (2014) reported that *Streptomyces flavotricini* isolated from Egyptian rice field soils showed the strongest antifungal activity against *M. oryzae*; the antifungal compound produced by *S. flavotricini* was successfully purified and identified as dihydroxy viridiofungin (C_37_H_58_N_2_O_10_).

Based on these findings, *S. vinaceusdrappus, S. philanthi* RM-1-138, *S. griseofuscus, S. hygroscopicus, Streptomyces* isolate 339, and *S. flavotricini* are potential candidates for use as biocontrol agents against rice blast as they possess inhibitory activity against *M. oryzae* (**Table 1**). Based on the percentage of mycelial growth inhibition, *S. philanthi* RM-1-138 appears to be one of the most promising agents for the inhibition of *M. oryzae*. However, studies involving greenhouse or field experiments are still required to more definitively evaluate the biocontrol potential of these *Streptomyces* spp. against *M. oryzae*.

**Table 1:** Summary of studies applying different *Streptomyces* strains as biocontrol agents for the control of rice blast disease caused by *Magnaporthe oryzae*.

Biocontrol agent	Type of experiment	Application method	Formulation	Results (percentage inhibition of *M. oryzae*)	Reference
*Streptomyces* strain BG2-53	Growth chamber experiment	Foliar spraying	Liquid (broth)	*Streptomyces* BG2-53 showed highest fungal control (98%) than Blasticidin-S (86%) and Tricyclazole (96%)	Lee et al., 2002
Endophytic *Streptomyces* (*S. griseofuscus, S. hygroscopicus, S. globisporus, S. aureus, S. albosporus*)	*In vitro* experiment using dual culture method	Not available	Not available	*Streptomyces griseofuscus* and *Streptomyces hygroscopicus* exhibited strongest antagonism against *M. oryzae*	Tian et al., 2004
*S. vinaceusdrappus*	*In vitro* experiment using dual culture method	Not available	Not available	Mycelial growth inhibition of *M. oryzae* (53.5%)	Ningthoujam et al., 2009
*S. sindeneusis* isolate 263	*In vitro* experiment using dual culture method	Not available	Not available	Antifungal activity against *M. oryzae*	Zarandi et al., 2009
	Greenhouse experiment	Foliar spraying	Liquid (culture filtrates)	Rice plants treated with *M. oryzae* alone showed typical blast symptoms and 8% diseased leaf area; rice plants treated with *M. oryzae* plus *Streptomyces sindeneusis* isolate 263 showed 0.5% diseased leaf area	
*S. globisporus* JK-1	*In vitro* experiment using dual culture method	Not available	Not available	Mycelial growth inhibition of *M. oryzae* (42.9%)	Li et al., 2011
	Greenhouse experiment	Foliar spraying	Liquid (culture filtrates)	*Streptomyces globisporus* JK-1 treatment (88.3%) showed highest fungal control than Tricyclazole (79.4%), as compared to the inoculated control	
*Streptomyces* isolate 339	*In vitro* experiment using dual culture method	Not available	Not available	Antifungal activity against *M. oryzae*	Zarandi et al., 2013
*S. philanthi* RM-1-138	*In vitro* experiment using dual culture method	Not available	Not available	Mycelial growth inhibition of *M. oryzae* (88.73%)	Boukaew and Prasertsan, 2014
*S. flavotricini*	*In vitro* experiment using dual culture method	Not available	Not available	Antifungal activity against *M. oryzae* (40 mm inhibition zone)^∗^	Khalil et al., 2014

*^∗^The percentage of inhibition of *M. oryzae* cannot be estimated due to insufficient information reported in the study.*

### Greenhouse/Growth Chamber Experiments

The limitation of laboratory experiments is that they only prove the antagonistic activity of *Streptomyces* spp. against *M. oryzae* under certain conditions. The antagonism exhibited by *Streptomyces* in laboratory experiments might not necessarily reflect antagonism under greenhouse or field experiments. Greenhouse or growth chamber experiments are conducted for the purpose of further evaluating the efficacy of *Streptomyces* strains as biocontrol agents. For *S. sindeneusis* isolate 263 and *S. globisporus* JK-1, studies on their biocontrol potential against the fungus under greenhouse conditions were conducted by Zarandi et al. (2009) and Li et al. (2011) respectively. Zarandi et al. (2009) reported that typical blast symptoms were observed when rice plants at the three leaf-stage of vegetative phase were treated with *M. oryzae*. The percentage of diseased leaf area was evaluated according to the method developed by the International Rice Research Institute (IRRI). It was found that the rice plants receiving treatment with *S. sindeneusis* isolate 263 showed significantly reduced lesion development. The diseased leaf area was 8% for rice plants treated with *M. oryzae* alone, while it was only 0.5% for rice plants treated with *S. sindeneusis* isolate 263 in combination with *M. oryzae*. This result indicates that *S. sindeneusis* isolate 263 acted as an antagonist.

Li et al. (2011) compared the control of the rice blast using *Streptomyces* and fungicide by infecting rice plants at the five leaf-stage during the vegetative growth phase with *M. oryzae*, followed by treatment with culture filtrates of *S. globisporus* JK-1 and tricyclazole respectively. Tricyclazole is one of the commonly used fungicides for the control of rice blast disease with several advantages over other fungicides, for instance, it is systemic in rice for blast control and has long residual effectiveness (Froyd et al., 1976). The results showed that control efficacy for *S. globisporus* JK-1 treatment was 88.3% and for tricyclazole was 79.4%, compared to the inoculated control. This suggests that *S*. *globisporus* is as efficient and possibly even superior to tricyclazole, with the additional benefits of biocontrol agents as compared to synthetic agents described earlier.

Additionally, a novel *Streptomyces* strain, BG2-53, with 96% homology to *S. lipmanii* based on analysis of 16S rDNA sequences, exhibited potent antifungal activity against *M. oryzae* under growth chamber conditions (Lee et al., 2002). The strain showed the highest degree of fungal control in comparison to fungicides such as Blasticidin-S and Tricyclazole. However, the evaluation on the extent of rice disease infection was solely based on visual estimation, unlike other studies where the Standard Evaluation System of IRRI was applied.

Overall, the results of greenhouse and growth chamber experiments strongly suggest that several *Streptomyces* spp. possess antagonistic activities against *M. oryzae*, and therefore have the potential to effectively control rice blast. Field experiments are still required to more definitively estimate the efficacy of *Streptomyces* spp. as biocontrol agents under real life conditions as environmental factors greatly affect their performance.

### Bioactive Compounds from *Streptomyces* spp. with Antifungal Activity against *M. oryzae*

The suppression of rice blast in the greenhouse by certain *Streptomyces* might indicate the presence of bioactive compound(s) with antifungal activity against *M. oryzae* in the culture filtrates. *Streptomyces* are prolific producers of bioactive compounds. Some well-known antibiotics produced by *Streptomyces* have been used as fungicides for the control of rice blast, for instance, Blasticidin-S and Kasugamycin. Blasticidin-S, isolated from *S. griseochromogenes* was the first antibiotic commercially introduced for the control of rice blast in Japan (Fukunaga et al., 1955; Takeuchi et al., 1958; Tapadar and Jha, 2013). Kasugamycin was discovered soon after; it was first isolated from *S. kasugaensis* by Umezawa et al. (1965). Kasugamycin has been safely used to protect rice plants against blast disease; it has relatively low mammalian toxicity and no phytotoxicity toward rice plants and most crops (Yamaguchi, 1982; Copping and Duke, 2007). Recently, the significance of Blasticidin-S as a fungicide has decreased as it has been replaced by new pathogen-specific fungicides with lower toxicity (Copping and Duke, 2007). Kasugamycin is currently still on the market and is sold in several different formulations such as wettable powder, granule, and soluble liquid under the trade names Kasumin and Kasumin-Bordeaux from Hokko Chemical Industry, Co., Ltd (Copping and Duke, 2007; Hokko, 2015).

Several studies have performed testing of the various compounds produced by *Streptomyces* on *M. oryzae*. These studies have shown that several compounds produced by *Streptomyces* spp. exhibited antifungal activity against *M. oryzae* (Appendix 1). The antibiotic Oligomycin A was first isolated from *S. diastatochromogenes* and was found to be active against several other phytopathogenic fungi in addition to *M. oryzae* such as *Botrytis cinerea, Cladosporium cucumerinum, Colletotrichum lagenarium, Phytophthora capsici, Alternaria alternata*, and *Aspergillus niger* (Smith et al., 1954; Kim et al., 1999; Yang et al., 2010). Oligomycin A’s ability to control the development of rice blast was evaluated in the greenhouse and the results showed that rice plants treated with Oligomycin A (50 μg/mL) had reduced lesions. When the concentration of Oligomycin A was increased up to 500 μg/mL, the rice plants did not show any rice blast disease symptoms (Kim et al., 1999).

Rapamycin—also known as Sirolimus—was initially isolated from *S. hygroscopicus* (Sehgal et al., 1975; Sehgal, 1998). Rapamycin is a potent antifungal agent found to be effective against *Candida albicans, Microsporum gypseum*, and *Trichophyton granulosum* (Sehgal et al., 1975). Bastidas et al. (2012) reported that Rapamycin inhibited the growth of *Mucor circinelloides* and *Rhizopus oryzae*, both of which are post-harvest fruit pathogens (Johnson et al., 1990; Kwon et al., 2011).

Other compounds such as Pyrroles (Pyrrolo[1,2-a] pyrazine-1,4-dione, hexahydro-) are commonly found in various *Streptomyces* species (Robertson and Stevens, 2014; Ser et al., 2015b, 2016b,c; Tan et al., 2015; Awla et al., 2016). Furthermore, Pyrrolo[1,2-a] pyrazine-1,4-dione, hexahydro-3-(phenylmethyl) was found to be able to protect plants from phytopathogenic fungi (Zhou et al., 2015).

Bioactive compounds derived from *Streptomyces* can be used for the management of fungal plant diseases as an alternative to synthetic fungicides (Prabavathy et al., 2006). These natural compounds are biologically synthesized and often biodegradable. Thus, they may be useful for the development of agricultural fungicides that are more pathogen-specific with minimal side-effects toward the environment (Varma and Dubey, 1999; Prabavathy et al., 2006). In order to formally establish any organism as a commercially viable biocontrol agent, studies involving the application of the microbial antagonist in question on a certain plant for the control of a particular pathogen are required to investigate their effectiveness. Although it is too early to draw any definite conclusions, the *Streptomyces* spp. listed in **Table 1** could be considered potential biocontrol agents, as they are capable of producing compounds with antifungal activity against *M. oryzae*.

## Antagonistic Mechanisms Of Biocontrol Agents

It is known that antagonistic activities of bacteria against fungal pathogens can be achieved through three main mechanisms: competition for nutrients and space, antibiosis, and parasitism (Gonzalez-Franco and Robles-Hernandez, 2009; Boukaew and Prasertsan, 2014). The advantages of *Streptomyces* spp. include their ability to colonize plant root surfaces, survive in various types of soil and also produce spores which allow them to survive longer and in various extreme conditions (Gonzalez-Franco and Robles-Hernandez, 2009; Ningthoujam et al., 2009). Antibiosis happens when the antagonist present in the plant produces metabolites such as antibiotics or antifungals which can inhibit or kill the pathogen. *Streptomyces* spp. used as biocontrol agents produced antibiotics such as geldanamycin and nigericin for the control of plant diseases, which were proven by the presence of antibiotics in soil (Rothrock and Gottlieb, 1984; Trejo-Estrada et al., 1998). Likewise, hyperparasitism may occur due to the release of extracellular lytic enzymes such as chitinases and glucanases from the biocontrol agent (Gonzalez-Franco and Robles-Hernandez, 2009; Palaniyandi et al., 2013). It has also been shown that *Streptomyces* spp. are capable of producing chitinases and glucanases which play important roles in destruction of fungal cell walls (Mahadevan and Crawford, 1997; El-Tarabily et al., 2000; Gonzalez-Franco and Robles-Hernandez, 2009). The colonization ability and competitive traits of *Streptomyces* could result in successful competition against phytopathogenic fungi and suppression of their growth. It can be presumed that the suppression of rice blast by *Streptomyces* spp. might be due to these mechanisms, but further studies are required to provide evidence regarding the actual biocontrol mechanisms of *Streptomyces* against *M. oryzae*.

## Challenges for Successful Application of Biocontrol Agents

**FIGURE 1:**
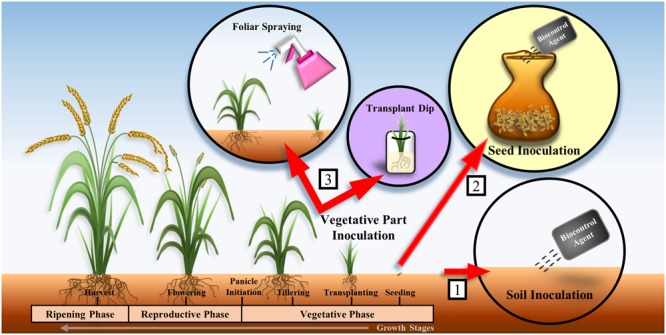
**Methods of application of biocontrol agents on rice plants: (1) soil inoculation, (2) seed inoculation, and (3) vegetative part inoculation.** Many studies involved the application of biocontrol agents on rice plant during the vegetative phase (Yang et al., 2008; Laborte et al., 2012; Gopalakrishnan et al., 2014).

One of the major challenges encountered during the selection of biocontrol agents is that biocontrol agents that appear efficacious based on *in vitro* experiments might not be effective in controlling plant diseases in greenhouse or field conditions. The efficacy of biocontrol agents is affected by organic matter, pH, nutrient level, and moisture level of the soil. Owing to the variations in environmental conditions in different locations, biocontrol agents that perform well in *in vitro* conditions might fail in greenhouse or field experiments. Therefore, the environmental factors at the location where biocontrol agents will be applied should be taken into consideration during the selection of appropriate biocontrol agents. Ideally, the biocontrol agents should be isolated from and applied to locations with similar environmental factors in order to achieve successful biological control (Suprapta, 2012). Furthermore, the formulation (e.g., powder, liquid, or granule) and the method of application of biocontrol agents such as soil inoculation, seed inoculation, and vegetative part inoculation should be examined (**Figure 1**) as they are important in determining the outcomes of field experiments (Ou, 1980; Dubey, 1993). Soil inoculation involves mixing of the biocontrol agent with soil or spreading the biocontrol agent in sowing furrows by drip systems (Vasudevan et al., 2002). Seed inoculation involves dipping seeds in a culture of the biocontrol agent or mixing the seeds with the inoculant using appropriate wetting agents (Dubey, 1993; Vasudevan et al., 2002; Yang et al., 2008). Vegetative part inoculation involves aerial/foliar spraying of the biocontrol agent or seedling treatment by dipping the roots of the seedlings into a solution containing the biocontrol agent prior to transplantation (Vasudevan et al., 2002; Gopalakrishnan et al., 2014) (**Figure 1**). The appropriate application method is likely to contribute significantly to the success of the biocontrol agents in the field trials (Suprapta, 2012).

## Examples of Commercial *Streptomyces* Biocontrol Agents

Some *Streptomyces* spp. have been successfully developed into commercial biocontrol agents and tested for the control of other plant diseases. For example, *Streptomyces griseoviridis* strain K61 (Mycostop^®^), which has been tested for the control of *Ceratocystis radicicola* that causes black scorch on date palm and soilborne pathogens of tomato such as *Fusarium oxysporum* f.sp. *lycopersici* and *Verticillium dahliae* (Suleman et al., 2002; Minuto et al., 2006). Other commercial *Streptomyces* biocontrol agents include *S. lydicus* WYEC108 (Actinovate^®^, Micro108^®^, Action Iron^®^), and *S. saraceticus* KH400 (YAN TEN *S. saraceticus*) (Elliott et al., 2009; Palaniyandi et al., 2013). Biocontrol agents are relatively safe toward humans as no adverse effects in users and other workers have been reported following exposure to these commercial products (Pest Management Regulatory Agency, 2003; U. S. Environmental Protection Agency, 2005). However, hypersensitivity may occur in certain individuals on exposure to biological dust produced during handling of *S. griseoviridis* strain K61 (Mycostop^®^) dry end-product (Pest Management Regulatory Agency, 2003). Hence, wearing appropriate safety equipment is required when handling these agents. With reference to the control of rice blast disease, however, commercial *Streptomyces* biocontrol agents have yet to be developed.

## Conclusion

Rice blast, the result of infection by *M. oryzae*, is the most destructive disease of rice, leading to crop yield losses and economic damage. While chemical control has been the mainstay of controlling this infection, biological control has now been introduced as an alternative for the management of rice blast disease. Biological control of plant diseases is typically inexpensive, long lasting, and safe toward the environment and living organisms; however, biological control can be a slow process and the search for suitable biocontrol agents requires considerable time and effort. *Streptomyces* spp. certainly demonstrate the potential to be developed as biocontrol agents due to their various beneficial properties. Based on current research findings, *S. vinaceusdrappus, S. philanthi* RM-1-138, *S. griseofuscus, S. hygroscopicus, Streptomyces* isolate 339, and *S. flavotricini* showed antifungal activity against *M. oryzae* under *in vitro* conditions. *S. sindeneusis* isolate 263 and *S. globisporus* JK-1 demonstrated *in vitro* antifungal activity against *M. oryzae* as well as successful biocontrol of rice blast in greenhouse experiments. *Streptomyces* strain BG2-53, which appears to be a novel strain, showed antifungal activity against *M. oryzae* under growth chamber conditions. These *Streptomyces* spp. possess antagonistic activities against *M. oryzae*, with *S. globisporus* JK-1 showing high control efficacy of up to 88.3%. Furthermore, studies have revealed that *Streptomyces* produces various compounds with antifungal activity against *M. oryzae.* Therefore, they are excellent candidates as biocontrol agents for the biological control of this devastating rice blast disease. In order to establish *Streptomyces* as biocontrol agents, more field experiments should be conducted to determine their control efficacy under different environmental conditions. Additionally, more work is needed to optimize isolation, formulation and application methods of *Streptomyces* in order to fully maximize their potential as effective agents to control rice blast.

## Author Contributions

The literature review and manuscript writing were performed by JL and H-LS, while TK, L-HC, PP, K-GC, B-HG, and L-HL provided vital guidance and insight for the writing. The research topic was conceptualized by L-HL and B-HG.

## Conflict of Interest Statement

The authors declare that the research was conducted in the absence of any commercial or financial relationships that could be construed as a potential conflict of interest.

## Footnotes

1 http://ricepedia.org/rice-as-food/the-global-staple-rice-consumers

2 http://www.bacterio.net/
