# Supplementary material for: The Potential of Streptomyces as Biocontrol Agents against the Rice Blast Fungus, Magnaporthe oryzae (Pyricularia oryzae)
Source: Front Microbiol. 2017 Jan 17;8:3. doi: 10.3389/fmicb.2017.00003 (PMC5239798; doi:10.3389/fmicb.2017.00003)
Supplement: Supplementary file 1 [file Data_Sheet_1.docx]

**Appendix 1. Bioactive compounds derived from different strains of *Streptomyces* with antifungal activity against the rice blast fungus *Magnaporthe oryzae***

| Author (Year) | Strain | Compound |
| --- | --- | --- |
| Iwasa et al. (1977) | *Streptomyces novoguineensis* sp. nov. | Amipurimycin |
| Kim et al. (1999) | *Streptomyces libani* strain As1 | Oligomycin A |
| Rhee (2003) | *Streptomyces* sp. KH-614 | Cyclo(L-Leucyl-L-prolyl) |
| Prabavathy et al. (2006) | *Streptomyces* sp. PM5 | SPM5C-1 (lactone carbonyl unit) |
|  |  | SMP5C-2 (ketone carbonyl unit) |
| Zhang et al. (2013) | *Streptomyces canus* BYB02 | Resistomycin |
|  |  | Tetracenomycin D |
| Xiong et al. (2013) | *Streptomyces padanus* JAU4234 | Antifungalmycin 702 |
| Khalil et al. (2014) | *Streptomyces flavotricini* | Dihydroxy viridiofungin |
| Awla et al. (2016) | *Streptomyces* sp. isolate UPMRS4 | Pyrrolo[1,2-a] pyrazine-1,4-dione, hexahydro-3-(2-methylpropyl) |
|  |  | Pyrrolo[1,2-a] pyrazine-1,4-dione, hexahydro-3-(phenylmethyl) |
|  |  | Ergotamine |
|  |  | Amicoumacin |
|  |  | Fungichromin |
|  |  | Rapamycin |
|  |  | N-acetyl-D |
|  |  | L-phenylalanine |
